# Content Determination of Active Component in Huangqi Yinyanghuo Group and Its Effects on hTERT and Bcl-2 Protein in Osteosarcoma

**DOI:** 10.1155/2014/769350

**Published:** 2014-10-13

**Authors:** Ying Tan, Lei Tan, Shuai Huang, Junfan Lu, Longtan Yu

**Affiliations:** ^1^Department of Spinal Surgery, Weifang Traditional Chinese Medicine Hospital, Weifang 261041, China; ^2^Department of Orthopaedic Surgery, The Sixth Affiliated Hospital of Sun Yat-Sen University, Guangzhou 510655, China

## Abstract

To screen the optimal extraction process and content determination of active component of Huangqi Yinyanghuo group (HYG) and to study the effects of HYG on human telomerase reverse transcriptase (hTERT) and Bcl-2 protein in osteosarcoma (HOS) cells, providing the theoretical basis for clinical application of HYG in treatment of osteosarcoma, orthogonal design table L_9_(4^3^) was used to design the extraction process of HYG, and icariin was taken as the investigation index to optimize the extraction process of HYG. 0.125, 0.25, 0.5, 1, 2, 4, and 8 *μ*mol/L HYG were taken to act separately on logarithmic growth phase osteosarcoma HOS cells, CCK-8 assay was used to determine cell viability, and immunohistochemical SP assay was used to determine the expression of hTERT and Bcl-2 protein. Apoptosis rate was positively correlated with the dose of HYG, and the expressions of hTERT and Bcl-2 protein were significantly decreased with the prolonged duration of action. Under the effect of HYG, dose was negatively correlated with osteosarcoma cell survival fraction; osteosarcoma cell survival fraction was positively correlated with hTERT and Bcl-2 protein; duration of action was negatively correlated with hTERT and Bcl-2 protein; and hTERT and Bcl-2 protein were in a synchronous relationship.

## 1. Introduction

Huangqi and Yinyanghuo are both healthy energy supporting traditional Chinese medicines. Huangqi can tonify Qi, and Yinyanghuo can support Yang; the two are commonly used in Chinese clinical medicine in the treatment of Qi deficiency, Yang insufficiency, and other diseases. They can also promote osteoblast function, increase calcified bones, and promote DNA synthesis of bone marrow cells; an in vitro experiment has shown that Yinyanghuo can promote osteoblast proliferation and differentiation [1]. Huangqi Yinyanghuo group (HYG) is the traditional Chinese medicine which is used in clinical practice widely [2, 3].

Telomerase plays an important role in the occurrence and development of tumor cells. Over 90% of tumor cells have telomerase activity, while the majority of somatic cells lack or even do not have telomerase activity [4]. Telomerase synthesizes telomeric DNA repetitive sequences by reverse transcription with its own RNA as the template and adds them to the telomere ends, in order to compensate for the terminal bases lost due to cell division and ensure the stability and integrity of chromosomes, thus providing the basis for eternal life of tumor cells. hTERT is its rate-limiting component, expression of hTERT genes plays a decisive role in telomerase expression, and Bcl-2 gene (i.e., B-cell lymphoma/leukemia-2 gene) is a type of protooncogene which is capable of suppressing apoptosis. hTERT and Bcl-2 protein are both very good targets for cancer therapy.

By studying the effects of HYG on hTERT and Bcl-2 protein in osteosarcoma cells, this experiment provides a theoretical basis for clinical application of HYG in treatment of osteosarcoma.

## 2. Instruments and Drugs

### 2.1. Instruments

Agilent 1100 series HPLC system; KQ-300 ultrasonic cleaner, Nanjing Kunshan Ultrasonic Instruments Co., Ltd.

### 2.2. Drugs

Huangqi and Yinyanghuo crude drugs (proportion of the two herbs was 1 : 1 in this compounded prescription) were purchased from Guangzhou Kelaimente Medicine Company, which were identified by the Institute of Botany of the Guangzhou Academy of Sciences as the dried root of* Astragalus membranaceus *(Fisch) Bge. var.* mongholicus* (Bge.) Hsiao of the family Leguminosae and* Epimedium brevicornum *Maxim. of the family Berberidaceae. Icariin reference substance (batch number 130526-152369) was obtained from Guangzhou Institute of Biological Products Assay; the water used was double distilled water, all the detection reagents were of HPLC grade, and other reagents were all of chemical grade.

Human osteosarcoma cell lines (HOS and CCK8) were purchased from Nanjing Dingsheng Biotechnology Co., Ltd. Fetal bovine serum (HyClone) was purchased from Beijing Saiaoruite Biochemical Products Co., Ltd. hTERT antibody was purchased from Santa Cruz Biotechnology; Bcl-2 antibody, DAB chromogenic reagent kit, and immunohistochemical staining kit (SP kit) were all purchased from Chengdu Hengyuan Bioengineering Co., Ltd.

## 3. Methods

### 3.1. Orthogonal Test Design

Investigation was performed on three levels of four factors: amount of water addition (*A*), soaking time (*B*), extraction time (*C*), and extraction times (*D*) by orthogonal design table L_9_(3^4^) using icariin as the indicator. See Table 1.

## 4. Determination of Icariin Content

### 4.1. Chromatographic Conditions

The chromatographic column used was Hypersil ODS2 (4.6 mm × 250 mm, 5 *μ*m), mobile phase was methanol-0.38% phosphoric acid solution (48 : 52), flow rate was 1.0 mL/min, detection wavelength was 270 nm, column temperature was 40°C, and sample injection volume was 10 *μ*L. The retention time of icariin was 10.2 min. The sample can be relatively well separated under these conditions.

### 4.2. Preparation of Test Solution

Icariin reference substance was accurately weighed and added to 70% methanol solution to prepare 0.01 mg/mL icariin reference solution; after filtration, 2.00 mL of filtrate was precisely drawn, placed in a 50 mL flask, added to 40 mL of 70% methanol solution, ultrasonicated for 10 min, allowed to cool and stand at room temperature, then diluted to the mark by addition of 70% methanol solution, shaken uniformly, and filtered with 0.45 *μ*m membrane, and the subsequent filtrate was collected as the test solution.

### 4.3. Plotting of Standard Curves

**Figure 1:**
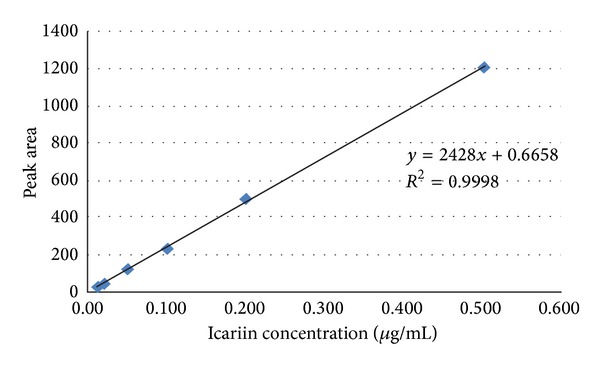
The standard curve of icariin.

Reference solution was taken and prepared into solutions with concentrations of 0.010, 0.020, 0.050, 0.100, 0.200, and 0.500 *μ*g/mL, respectively; 10 *μ*L of the solutions was taken, and their peak areas were measured; standard curves were plotted with the peak area as ordinate (*Y*) and the icariin concentration as abscissa (*X*), and regression equation was calculated to be *Y* = 2428*X* − 0.6658, *R* ^2^ = 0.9999. The results showed that the icariin concentration had a good linear relationship with the peak area within the concentration range of 0.01~0.50 *μ*g/mL; see Figure 1.

### 4.4. Precision Test

10 *μ*L of reference solution was precisely drawn, sample was injected 5 times in parallel, peak area value of icariin was measured, and RSD value was calculated to be 1.52%, which indicated good precision of the instrument.

### 4.5. Stability Test

Test solution was prepared according to the test solution preparation method, sample was injected after standing for 0, 2, 4, 8, and 12 h, respectively, peak area of icariin was measured, and RSD value resulted to be 2.50%, which indicated that the test solution had good stability within 12 h.

### 4.6. Reproducibility Test

Test solutions were prepared in quintuplicate according to the test solution preparation method, and peak areas were measured; RSD of content was calculated to be 1.83%, which indicated good reproducibility of the present method.

### 4.7. Sample Recovery Test

Approximately 0.20 g of samples with known concentration was accurately weighed in quintuplicate, placed in stoppered Erlenmeyer flasks, and added precisely with 2 mL of 0.198 mg/mL icariin solution in dilute ethanol, and peak area was measured according to the method under sample assay, content was determined, and average recovery rate resulted to be 100.5%, with RSD value of 1.72%.

### 4.8. Orthogonal Test

Crude drugs were extracted according to the orthogonal design table L_9_(3^4^), extracts were combined, appropriate volume of extract was taken, and icariin content in the extract was determined according to the method under “plotting of standard curves”; the results and analysis are shown in Tables 2 and 3.

It can be judged according to the results in Table 2 that the size of influence of various factors on icariin extraction was *D* > *A* > *B* > *C* in descending order; optimal extraction process was *A*_3_ *B*_1_ *C*_2_ *D*_3_, that is, addition of a 12-fold volume of water to the Quangqi and Yinyanghuo crude drugs with a weight ratio of 1 : 1, soaking for 15 min, and three times of extraction with each time lasting 1.0 h; the residues were then discarded and the decoctions were combined to give the extract. As can be seen from Table 3, factor *D* has a significant influence on extraction of icariin, while factors *A*, *B*, and *C* have relatively small influences within the investigation range, which presented no statistical significance.

### 4.9. Validation Test

The above screened optimal extraction process was validated, and the validation results are shown in Table 4. According to the results in Table 4, average content of icariin in the extract is 69.07 *μ*g, which is higher than the content in any test number, and does not have relatively large deviation as well.

### 4.10. Cell Culture

Cells were subcultured in an incubator set at 37°C, 5% CO_2_, and saturated humidity; subculture medium was 10% FBS and 100 *μ*/mL double antibody-containing high glucose DMEM (containing phenol red).

### 4.11. CCK-8 Detection of Cell Proliferation

Cells were seeded in 96-well plates at 5 × 10^3^ cells per well. After culturing for 24 h, each well was filled separately with 200 *μ*L of HYG-containing media with final concentrations of 0.125, 0.25, 0.5, 1, 2, 4, and 8 *μ*mol/L; meanwhile, a nondrug treatment group was set up; after incubation it was continued in the incubator for additional 24 h, 48 h, 72 h, 96 h, and 120 h, and each well was filled with 20 *μ*L of CCK-8 solution and incubated in the incubator for another 4 h. Absorbance of each well was measured at 490 nm wavelength using ELISA reader; cell morphology was observed under a microscope and photographed. Cell growth curve was plotted with time as the horizontal axis and absorbance as the vertical axis. Survival fraction (SF) was calculated as follows: SF (%) = (OD value of experimental group/OD value of control group) × 100%. The experiment was repeated three times in parallel; finally, time-survival curves were plotted according to the SF mean ± standard deviation $\left(\overset{-}{x}\pm s\right)$ of three samples. 2.7 immunohistochemical detection of hTERT and Bcl-2 protein expressions.

Cells were seeded in 24-well plates at 4 × 10^5^ cells per well, two experimental groups were set up, and each group contained three parallel samples; meanwhile, a control group was set up. After culturing for 24 h, each well was filled with 1 mL of medium containing optimal concentration of HYG and then cultured for 3 d and 5 d. Streptavidin peroxidase (SP) method was used. Cells were fixed conventionally and stained, and the ones with appropriate morphological characteristics were selected under a 10x inverted microscope and photographed. Semiquantitative analysis of immunohistochemical images were performed using Image J and Image Pro Plus software.

### 4.12. Statistical Analysis

Statistical processing: statistical processing was performed using SPSS software. Data were expressed as $\left(\overset{-}{x}\pm s\right)$, comparison among groups was performed by* t-*test, and correlation analysis was performed by paired chi-square test; *P* < 0.05 was considered statistically significant.

## 5. Results

### 5.1. Cell Survival after Action of HYG

**Figure 2:**
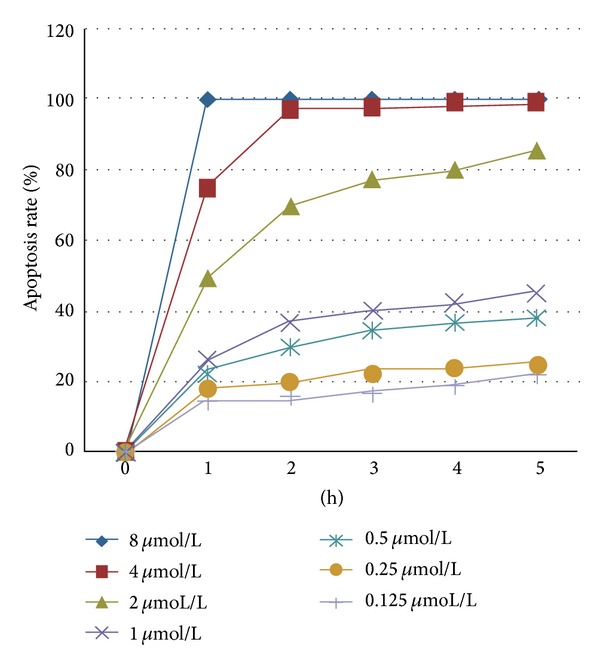
Relationship of apoptosis rate with time and dose.

**Figure 3:**
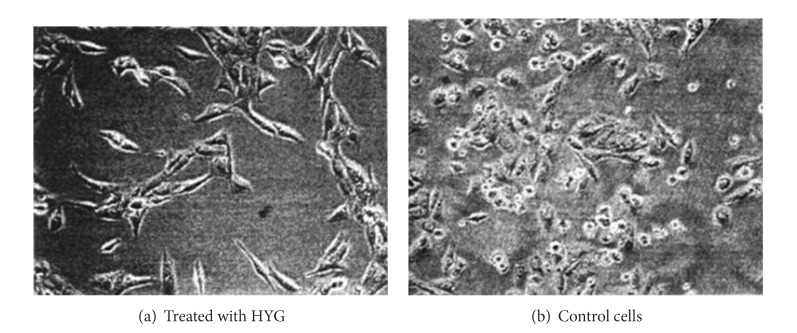
The morphological changes of osteosarcoma.

Cell survival curve describes the survival status of osteosarcoma cells at different doses and times; see Figure 1. As can be seen from Figure 2, the survival fraction of osteosarcoma HOS cells decreases markedly with the passage of time, and no significant late stage cell proliferation is present. Correlation analysis showed that the cell survival fraction was negatively correlated with the drug dose (*P* < 0.01). The morphological changes of osteosarcoma, after HYG treatment, and untreated osteosarcoma cells, under light microscopy, form as shown in Figure 3.

### 5.2. hTERT and Bcl-2 Protein Detection

**Figure 4:**
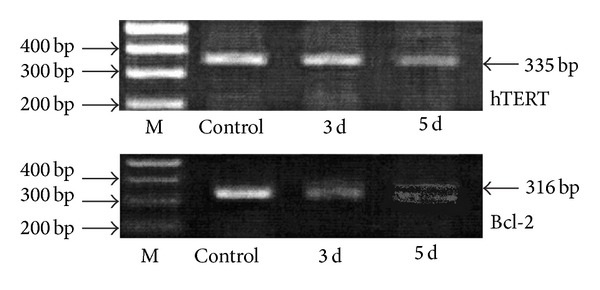
The mRNA expression of hTERT and Bcl-2.

Immunohistochemical images were analyzed using Image Pro Plus software; the results showed a gradual increase in gray scale value of each image, which indicated gradual decrease in protein content of each image. The data showed that the hTERT and Bcl-2 protein expressions in the experimental groups were apparently lower than the control group and that the expression intensity was negatively correlated with time (Figure 4 and Table 5).

Positive cell counting was performed on immunohistochemical images using Image J software; the higher the count of positive cells, the larger the number of cells expressing hTERT and Bcl-2 protein, which also means wide distribution of hTERT and Bcl-2 protein-expressing cells, and vice versa. The difference was significant by paired chi-square test (*P* < 0.05) (Table 6).

### 5.3. Relationship between hTERT and Bcl-2 Protein Expressions

The paired chi-square test found that hTERT and Bcl-2 protein expressions have a high degree of consistency and a significant correlation (*P* < 0.05).

## 6. Discussion

### 6.1. Selection of Investigation Indices

Icariin is one of the main active ingredients in Yinyanghuo; pharmacological studies have shown that icariin has multiple pharmacological activities such as antitumor, immunity enhancement, cardiovascular function improvement, and endocrine regulation [5]. Many TCM preparations with Yinyanghuo as the monarch drug [6, 7] used icariin as the investigation index; therefore, the selection of icariin as the investigation index for screening of extraction process in this study is feasible and reasonable.

### 6.2. Selection of Chromatographic Conditions

Referring to the literature, the author also tried to select different mobile phases, acetonitrile-water [8], methanol-acetic acid [9], methanol-water-glacial acetic acid [10], and so forth, which resulted in poor separation effects at the position of icariin peak, with interferences from other small peaks; when methanol-0.38% phosphoric acid solution (48 : 52) was used, icariin peak separated well from other impurity peaks, without interfering peaks.

Through the understanding of many behavioral relationships between hTERT and Bcl-2 protein, this experiment aims to understand the effects of HYG on hTERT and Bcl-2 proteins in osteosarcoma (HOS) cells and possible correlation; meanwhile, it provides a theoretical basis for clinical application of HYG.

Osteosarcoma (osteogenic sarcoma) is the most common malignant bone tumor. Typical osteosarcoma usually occurs in the 10–20 age group; nevertheless, it may occur in any age group, including infancy, childhood, and old age. Men have a higher incidence than women, with a male-to-female ratio of approximately 2 : 1. Osteosarcoma has strong local infiltration and lung metastasis capacities. Surgical treatment has greatly improved the prognosis of patients, but disability rate remains very high.

Telomerase is expressed at a very high level in 90% of cancer cells, while it is rarely expressed in normal cells; it is a recognized anticancer target [11]. hTERT is the rate-limiting enzyme of telomerase [12], which regulates the activity of telomerase [13] and has a parallel relationship with telomerase activity [14, 15]. At present, many scholars have turned their eyes to hTERT, hoping to achieve a breakthrough for the treatment of cancer [16]. Bcl-2 gene is the focus of everyone's attention; there have been many reports that Bcl-2 protein can protect cells from apoptosis and increase the expression of hTERT [17–19], but there are also some experiments which demonstrated that Bcl-2 protein is not correlated with, or even negatively correlated with, hTERT [20, 21].

The present study found that untreated osteosarcoma cells were mainly in short spindle shape under light microscope and were arranged tightly, with large nuclei, nuclear division was common, and cells were grown in multilayers when they were dense. After treatment with HYG, cell morphology changed obviously, cells were mainly in polygonal shape and were arranged in a monolayer, cell size tended to be uniform, and nuclei were small and were deeply stained; these results indicated the occurrence of morphological differentiation and changes. Osteosarcoma cell apoptosis was significantly increased with the increase of drug concentration; cell apoptosis was also significantly increased with the extension of drug action time. Under the effect of optimal concentration, with time as the axis, hTERT and Bcl-2 protein expressions did not change significantly in the control group; in the experimental group 3 d and experimental group 5 d, hTERT and Bcl-2 protein expressions gradually weakened and were in a negative correlation with the time of drug action, which were consistent with relevant reports [22, 23]; the two were in a time-dependent relationship with the drug and showed a high degree of consistency with respect to expression.

The study results showed the presence of time dependence between HYG and hTERT but could not confirm that HYG was able to directly influence telomerase activity; there existed high degree of consistency between Bcl-2 protein and hTERT, but whether there exists mutual assistance between the two needs further research; due to the discovery of telomerase activity in part of normal tissues, whether HYG has toxic side effects on them also needs further study; there is also the possibility of false positive and false negative results in nonfull quantitative determination of hTERT and Bcl-2 gene protein expressions; tumor cells were in a low pH state [24], but the tumor cells in the present experiment were in a state where pH is 7.2–7.4, which cannot fully reflect the low pH state of malignant tumor tissues in vivo. The above issues need to be further demonstrated.

## Figures and Tables

**Table 1:** Factors and levels of orthogonal test.

Level	Factor			
	*A* (amount of water addition, folds)	*B* (soaking time, min)	*C* (extraction time, h)	*D* (extraction times, times)
1	8.0	15	0.5	1
2	10.0	30	1.0	2
3	12.0	45	1.5	3

**Table 2:** Orthogonal test results.

Test number	Factor				Icariin content in
	*A*	*B*	*C*	*D*	extract (*μ*g)
1	1	1	1	1	24.23
2	1	2	2	2	37.45
3	1	3	3	3	42.60
4	2	1	2	3	53.50
5	2	2	3	1	28.70
6	2	3	1	2	27.50
7	3	1	3	2	51.63
8	3	2	1	3	48.72
9	3	3	2	1	33.55
*K*1	34.77	43.10	33.45	28.82	
*K*2	36.55	38.40	41.47	38.86	
*K*3	44.61	34.50	40.89	48.23	
*R*	9.845	8.546	7.990	19.432	

**Table 3:** Analysis of variance.

Factor	Sum of squared deviations	Degree of freedom	*F* value	*F* critical value	*P* value
*A*	165.150	2	1.496	4.458	>0.05
*B*	110.610	2	1.000	4.458	>0.05
*C*	120.219	2	1.088	4.458	>0.05
*D*	566.489	2	5.112	4.458	<0.05

**Table 4:** Validation test results.

Test number	Icariin content in extract (*μ*g)	Average content
1	69.23	69.07
2	69.36	
3	68.37	
4	69.19	
5	69.22	

**Table 5:** Analysis of gray scale value of immunohistochemical images.

Group	Gray scale level	
	hTERT	Bcl-2
Control group	26.44 ± 28.23	27.23 ± 18.56
Experimental group (3 d)	38.26 ± 31.52	51.41 ± 19.62
Experimental group (5 d)	47.21 ± 27.23	60.25 ± 20.39

**Table 6:** Analysis of positive cell count of immunohistochemical images.

Group	hTERT		Bcl-2	
	Positive	Negative	Positive	Negative
Control group	50%	50%	72%	28%
Experimental group (3 d)	39%	61%	55%	45%
Experimental group (5 d)	25%	75%	27%	73%

## References

[1] Li FF, Song SP, Li JP, Li N (1999). Effects of, *Epimedium brevicornum* Maxim. on osteoblast proliferation and differentiation. *Chinese Journal of Osteoporosis*.

[2] Cao JY, Zhou HB, Lu XC, Dou SL, Liu YW, Li XB (2003). The effects of florfenicol and Chinese herbal ingredients radix astragali and Herba epimedii on humoral immune response in chicks. *Acta Veterinaria et Zootechnica Sinica*.

[3] Ran HM (2011). From draws up “Huang Qiyin Yang Huotang” treatment chronic nephritis. *Chinese Manipulation & Rehabilitation Medicine*.

[4] Kim NW, Piatyszek MA, Prowse KR (1994). Specific association of human telomerase activity with immortal cells and cancer. *Science*.

[5] Li L, Wang X-M (2008). Progress of pharmacological research on icariin. *China Journal of Chinese Materia Medica*.

[6] Lu M, Chen BH, Shen SJ (2008). Determination of Icariin in Bushen capsule. *Modern Chinese Medicine*.

[7] Ye F, Wang YH, Wang XQ (2008). HPLC determination of fluorouracil in lymph node. *Chinese Journal of Hospital Pharmacy*.

[8] Wang SH, Chen JP (2008). Study on the extraction process of Yinyangsuo chewable tablets by orthogonal design. *Chinese Journal of Modern Drug Application*.

[9] Han XM, Zheng H, Gao YF (2008). Determination of Icraiine content in xianling gubao tablets by HPLC. *Heilongjiang Medicine Journal*.

[10] Jin FH, Zhou XM (2008). Determination of icariine in Wangbi Granules by HPLC. *China Medical Herald*.

[11] Kido A, Tsujiuchi T, Morishita T (1998). Telomerase activity correlates with growth of transplantable osteosarcomas in rats treated with cis-diammine dichloroplatinum or the angiogenesis inhibitor AGM-1470. *Japanese Journal of Cancer Research*.

[12] Rama KS, Eppenber GV, Shin KH (1998). Expression profile of the putative catalgtic subunit of theTelomerase gene. *Cancer Research*.

[13] Arinaga M, Shimim S, Gotoh K (2000). Expression of human telomerase subunit genes in primary lung cancer and its clinical significance. *The Annals of Thoracic Surgery*.

[14] Sharma GG, Gupta A, Wang H (2003). hTERT associates with human telomeres and enhances genomic stability and DNA repair. *Oncogene*.

[15] Masutomi K, Yu EY, Khurts S (2003). Telomerase maintains telomere structure in normal human cells. *Cell*.

[16] Zhu Y, Zheng JL, Zhang B, Zhang QW, Li QZ (2003). Expression of human telomerase reverse transcriptase and c-myc in osteosarcoma. *Chinese Journal of Clinical Oncology*.

[17] Mandal M, Kumar R (1997). Bcl-2 modulates telomerase activity. *The Journal of Biological Chemistry*.

[18] Atsushi YA, Kazwo H (2000). Te-lomerase activity in colorectal cancer and its relationship to Bcl-2 expression. *Journal of Surgical Oncology*.

[19] Fu WN, Begley JG, Killen MW, Mattson MP (1999). Anti-apoptotic role of telomerase in pheochromocytoma cells. *The Journal of Biological Chemistry*.

[20] Vietor M, Winter S, Groscurth P, Naumann U, Weller M (2000). On the significance of telomerase activity in human malignant glioma cells. *European Journal of Pharmacology*.

[21] Ohmura Y, Aoe M, Andou A, Shimizu N (2000). Telomerase activity and Bcl-2 expression in non-small cell lung cancer. *Clinical Cancer Research*.

[22] Zhao Q, Yang Y, Yu J (2008). Posttranscriptional regulation of the telomerase hTERT by gambogic acid in human gastric carcinoma 823 cells. *Cancer Letters*.

[23] Xu XY, Liu YQ, Wang L (2009). Gambogic acid induces apoptosis by regulating the expression of Bax and Bcl-2 and enhancing caspase-3 activity in human malignant melanoma A375 cells. *International Journal of Dermatology*.

[24] Aghunand N, He X, Van SR (1999). Enhancement of chemo therapy by manipulation of manipulation of tumor PH. *British Journal of Cancer*.

